# Hypothermia in refractory status epilepticus

**DOI:** 10.1186/cc11284

**Published:** 2012-06-07

**Authors:** Andrea O Rossetti

**Affiliations:** 1 Service de Neurologie, CHUV-BH07, Lausanne, Switzerland

## Introduction

Status epilepticus (SE) is a neurological emergency with potentially important mortality and morbidity. After refractoriness to general anesthetics, several pharmacological and nonpharmacological options have been described more or less anecdotally. In this context, and despite animal data supporting neuroprotective actions of brain hypothermia and showing its efficacy in SE models, hypothermia targeting a core temperature of about 33°C for at least 24 hours together with pharmacological sedation has been scarcely reported in adults and children. It seems that this approach rarely allows a sustained control of SE, as seizures tend to recur in normothermic conditions. Conversely, hypothermia has a high evidence level and is increasingly used in postanoxic encephalopathy, both in newborns and adults. Due to the thin available clinical evidence, prospective studies are needed to define the value of hypothermia in SE.

## Refractory status epilepticus and its treatment

SE represents the second most frequent neurological emergency after acute stroke, and bears significant risks of morbidity and mortality [1]. SE persisting despite adequate doses of benzodiazepines and at least one antiepileptic drug (AED) is considered refractory (RSE) [2,3]; this develops in 23 to 43% of patients with SE. RSE is associated with acute, severe and potentially fatal underlying etiologies, such as encephalitis, large stroke, or rapidly progressive primary brain tumors, and may be accompanied by coma; these factors, together with increasing age, represent the most important outcome predictors [1].

After securing pulmonary and cardiac functions, intravenous administration of a sequence of three groups of drugs represents the mainstay of management [4]: benzodiazepines (the only clearly evidence-based step); classical antiepileptic drugs (AED, mostly phenytoin, valproate, or levetiracetam); and general anaesthetics for RSE. Among anaesthetics, midazolam, propofol, or barbiturates represents the most popular agents, without any hard evidence favoring one specific compound [1]. Anesthetic treatment may lead to various complications (infections, metabolic disturbances, ileus, neuropathy, myopathy, thromboembolic events) [5]; it is therefore necessary to balance these risks with the benefit of rapid seizure control. Generalized convulsive RSE should be treated rapidly with general anesthetics, given the danger of systemic and neurological injury with ongoing convulsions; conversely, nonconvulsive SE without marked consciousness impairment can be approached more conservatively, as these forms are probably not associated with the same risk of injury [1,2,6].

RSE that proves refractory to a first course of general anesthetics implies a (repeated) careful search of the underlying etiology. This condition may be managed in several ways, which mostly rely on small series or case reports [1,7]. Briefly, pharmacological options may include further use of anesthetics (the three aforementioned, ketamine, isofluorane), other AED (for example, topiramate, lacosamide), or ketogenic diet. Reported nonpharmacological approaches span from resective surgery, through vagus-nerve, electroconvulsive or transcranial magnetic stimulations, to mild therapeutic hypothermia (TH).

While the benefits of hypothermia on patients with head injury were already described by Hippocrates [8], TH enjoys an only evidence-based status in the setting of adult and pediatric postanoxic encephalopathy, and reduction of intracranial pressure [9]. Its indication for the treatment of other acute brain disorders, including SE and traumatic brain injury, is essentially anecdotic.

## Animal data on hypothermia

Low brain temperature exerts beneficial effects on the cascades involved in acute cerebral injuries; several seminal studies have been recently reviewed [9,10]. Hypothermia reduces brain metabolism and ATP consumption, and leads to decrease of glutamate release, free radicals, oxidative stress, mitochondrial dysfunction and calcium overload. Conversely, brain-derived neurotrophic factor increases; as a result, apoptosis is inhibited. Furthermore, hypothermia reduces reperfusion injury, permeability of the blood-brain barrier, and inflammatory reactions. Of note, most of these mechanisms are involved in the pathophysiology of SE leading to neuronal injury [11]. Various rodent models of SE support the neuroprotective effects of TH, showing (with concomitant benzodiazepines) a reduction of seizure severity in SE triggered by electrical stimulation of limbic structures [12], and mitigation of seizures, brain edema, and cognitive deficits in kainate-induced SE [13]. Temperature lowering before pilocarpine injections (a proconvulsant) protects against SE and apopotosis [14]. However, TH has received very little attention in clinical settings.

## Experience in patients with refractory status epilepticus

More than two decades ago, three children with generalized SE were successfully treated with TH (30 to 31°C) and thiopental [15]. Four adults with SE triggered by limbic encephalitis (two patients), hepatic encephalopathy (one patient) and of unknown origin (one patient) were treated with TH (31 to 35°C) co-administered with midazolam; SE was controlled in all, but two patients later died [16]. Shivering was managed by neuromuscular blockade; vein thrombosis and pulmonary embolism were the reported side effects. Another adult with cryptogenic SE was treated with TH (34°C) and thiopental, but she developed paralytic ileus requiring emergency intestinal resection; she survived after further treatment of her RSE [5]. Ongoing seizures in an infant with a severe developmental disorder were controlled by TH at 35 to 36°C, together with ketamine; subsequently, hemispherectomy had to be carried out [17]. Finally, an abstract in Japanese describes an improved functional outcome in 12 children treated with TH (34 to 36°C) and general anesthesia for febrile SE, as compared with 16 treated with conventional therapy, in a retrospective assessment [18]. Reported TH durations are variable, between 20 hours and several days. These case studies suggest that hypothermia may contribute to seizure control. However, its efficacy seems to be only transient: seizures tend to recur in normothermia. TH may thus represent an option in severely refractory SE, but rather to gain some time as to definitively control seizures.

Recently, it has been recognized that postanoxic SE, even with early myoclonus, does not imply an invariably dismal outcome. It seems that SE occurring during TH, mostly as a seizure suppression EEG pattern, does reflect an extremely severe brain damage, and patients are extremely unlikely to survive [19,20]; conversely, SE arising after return of normothermia and in presence of a reactive EEG background, and preservation of brainstem reflexes and early cortical somatosensory evoked potentials, may be successfully treated with the usual therapeutic armamentarium; those cases represent at most 10% of patients with postanoxic SE, and a good functional outcome can be reached [21]. This actually suggests that TH (with moderate midazolam or propofol doses) can be sufficient to transitorily control benign postanoxic SE (corroborating its antiepileptic properties), while it does not prevent a poor outcome in more severe forms.

## Conclusion

As there is a lack of clinical evidence, mild TH (32 to 36°C) may represent a therapeutic option for RSE, albeit on a patient by patient basis. Barbiturates should be avoided because of the risk of paralytic ileus (thus favoring midazolam or propofol), and mild hypothermia should be administered for 24 to 48 hours. Repeated controls of cardiovascular indices, coagulation parameters and lactate (metabolic acidosis following severe infections or intestinal necrosis), and clinical surveillance (vein thrombosis) are mandatory. A well-designed, prospective trial appears necessary to assess the exact role of TH in SE.
